# Synthesis and Biological Evaluation of a New Polymeric Conjugate and Nanocarrier with Osteotropic Properties

**DOI:** 10.3390/jfb3010079

**Published:** 2012-01-19

**Authors:** Rosario Pignatello, Maria Grazia Sarpietro, Francesco Castelli

**Affiliations:** Dipartimento di Scienze del Farmaco, Università degli Studi di Catania, viale A. Doria 6, Catania I-95125, Italy; E-Mails: mg.sarpietro@unict.it (M.G.S.); fcastelli@unict.it (F.C.)

**Keywords:** PLGA, alendronate, bone-seeking polymer, nanoparticles, biocompatibility, hemocompatibility, doxorubicin, osteolytic tumors, bone metastasis

## Abstract

Bone-seeking (osteotropic) drug delivery systems (ODDS) represent an interesting solution for targeting different types of drugs to the bones. In particular, anticancer and antibacterial agents could take advantage of such therapeutic strategy. We have recently developed an innovative approach to this aim: a new osteotropic biomaterial was prepared, based on the conjugation of a poly(lactide-*co*-glycolide) (PLGA) with the bisphosphonate drug alendronate (PLGA-ALE); its hemo- and cytocompatibility were verified. Starting with this copolymer, an osteotropic nanoparticle system (NP) was produced for the targeted delivery of antineoplastic drugs to osteolytic bone metastases; in particular, doxorubicin was tested as a model drug. The *in vitro* and *in vivo* results of the new ODDS are validated in this article. All the experimental data confirmed that the drug retained its activity after loading in the PLGA-ALE NP; they can be thus considered a new promising strategy for active targeting of drugs to bone tissues in different pathological situations.

## 1. Introduction

The clinical treatment of many cancers, including bone primary tumors or bone metastases would take great benefit from the selective release of anticancer drugs, *i.e.*, from active or passive targeting approaches [1]. Bone is the third most common site of metastasis from peripheral cancers [2] and they represent a serious and costly complication of many cancers. Primary carcinomas of breast, prostate, kidney, lung, and thyroid may in particular be the origin of skeletal metastases, as well as multiple myeloma. A possible explanation for this is the high blood flow that characterizes the bone and bone marrow, and that drives migrating cancer cells to these areas.

Metastatic bone disease is the consequence of the interaction between malignant and bone cells. These metastases can be defined as either osteolytic or osteoblastic. In the first case, the tumor provokes bone dissolution and erosion, accompanied by calcium loss. They are for instance characteristic of multiple myeloma. Osteoblastic bone lesions, conversely, show an increased bone production, resulting in the formation of a rigid, inflexible bone. Prostate cancer typically induces this kind of metastases; however, many cancers can lead to both types of lesions. In more than 80% of cases an increase of the osteoclast activity is observed (osteolytic metastases), offering a rational target for new therapeutic approaches [3]. Tumor-induced osteolysis causes skeletal pathologies such as bone fractures, intractable bone pain, nerve root and spinal cord compression syndrome, and a high-grade hypercalcemia [4]. Anemia and complications given by the immobilization often exacerbate the clinical framework, which normally displays an unfavorable prognosis and a great loss in quality of life [4].

In osteolytic metastases different factors are responsible for the activation of osteoclasts, depending on the type of tumor. Neoplastic cells themselves secrete adhesive molecules that can bind to the bone tissues. This interaction acts as a signaling for increased bone destruction and tumor development inside the bone [5,6]. Bone-colonizing tumor cells in fact induce the secretion of osteolytic agents; the consequent augmented bone resorption releases bone-derived growth factors into the systemic circulation, thereby further enhancing bone resorption, altering the tumor microenvironment and promoting the growth of the tumor mass [7]. This results in a self-generating cancer growth loop.

Clinical management of metastatic bone disease is actually difficult, but new perspectives could be opened by different innovative strategies [4]. Current treatments are based on both systemic therapy (chemotherapy, immunotherapy, hormone therapy), local surgery and radiotherapy [8,9].

The restoration of functionality and mobility of the osteo-articular apparatus, along with pain relief can improve patients’ quality of life, but is unable to change the prognosis of the disease. Due to the peculiar features of tumors cell growth inside the bone tissues, conventional therapies often are ineffective against metastases. A number of drugs are effective for the treatment of bone tumors, but their systemic administration is inexorably associated with important counter-effects and lack of selectivity. Thereby, targeting specific biochemical pathways inside bone cancer loci may theoretically furnish a tool to enhance the efficacy and safety of potentially valid anticancer agents. 

### 1.1. Approaches for Drug Targeting to Bone

Bone-seeking (osteotropic) drug delivery systems (ODDS) represent an intriguing solution for bone targeting of different types of drugs. 

**Figure 1:**
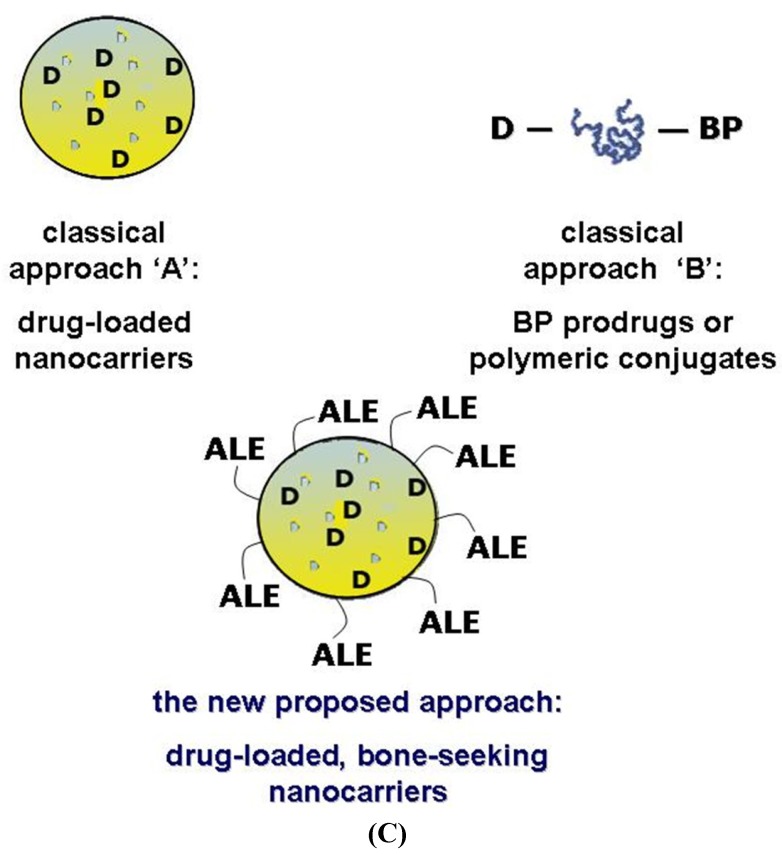
The possible strategies for selective delivering bioactive compounds to the bone: (**A**) a drug or diagnostic agent **D** is encapsulated/loaded into a conventional nanocarrier (e.g., polymeric or lipid nanoparticles, liposomes, micelles, *etc*.) and passive targeting is expected (EPR effect); (**B**) a drug is covalently linked to an osteotropic homing-moiety (e.g., a bisphosphonate, BP) or, a polymeric conjugate bearing the drug and the targetor is produced; (**C**) smart nanoparticles are produced with a bone-seeking polymer (e.g., the polymer-BP conjugate described in our studies) and then loaded with a bioactive agent; consequently, both passive and active targeting opportunities are achievable.

The strategies proposed up to now can be summarized in two main areas: passive and active targeting approaches. The first one can be attained by encapsulating in or associating drugs to colloidal vectors like liposomes, polymeric or lipid-based nanoparticles (NP), or dendrimers (Figure 1A). By drug-loaded nanocarriers a selective delivery can be obtained to specific tissues, like tumors, by taking advantage of their leaky neovasculature and absence of lymphatic drainage (“EPR effect”) [10]. 

Nano-sized carriers can be also engineered to show multifunctional properties; for instance, they can contain both a targeting group and active compound(s), thus being able to deliver the latter at specific cells or tissues. NP may possess the further advantage of maintaining poorly soluble or unstable compounds in the circulation, such as peptides and proteins, preventing their premature inactivation by plasma enzymes and prolonging their activity.

Active targeting strategies may be more successful to reach adequate drug concentrations at the target sites. Drugs and diagnostic agents can be covalently linked to a ‘targetor’ moiety able to recognize the bone cells, and selectively transport the whole compound (that is, a prodrug or a polymer conjugate) to its site of action (Figure 1B) [11]. 

Bones consist of a mineralized matrix; thereby, a rational approach for targeting drugs to these areas is to synthesize chemical delivery systems showing a certain affinity for hydroxyapatite (HA). This goal can for instance be attained by linking osteotropic compounds, such as bisphosphonates (BPs), to a bioactive compound or a drug carrier [12,13,14]. BPs are synthetic, non-hydrolysable compounds structurally related to pyrophosphate. The phosphorous-carbon-phosphorous (P-C-P) structure present in BP molecules allows the binding of metal ions, such as calcium, and explains the absorption to the HA domains of the bones [15]. When administered systemically BPs are rapidly cleared from the circulation and bind to mineral bone surface at the sites of active bone remodeling, like regions subjected to osteoclast resorption [15]. 

Clinically, BPs are potent antiresorptive agents used for treating different bone pathologies associated to an increase in the number and/or activity of osteoclasts, such as Paget’s disease and osteoporosis [16]. BPs are however also efficacious in reducing the proliferation, adhesion, migration, and invasion of tumor cells [17,18] and to inhibit the neo-vessel formation in experimental and animal tumor models [19]. The more recent BPs have become a valid supportive line of treatment for the relief of pain associated with either osteolytic and osteoblastic metastases [20]. In particular, they represent an alternative way of severe bone pain management for those patients who are not responsive to local radiotherapy [3].

Since HA crystals are only present in “hard” tissues, like bones and teeth, conjugation of drugs to a BP molecule can represent a valid strategy for their selective release to the bone surface. Many scientific publications and patents have been produced using this strategy, some of them presenting an original design and often merging different chemical and technological pathways. Among them, osteotropic drug delivery systems (ODDS) have been proposed as a tool to impart to drugs a certain affinity for bone tissues [12,13,21]. BP compounds have been conjugated to drugs [22,23,24], and proteins [25] with the aim of optimizing the treatment of osteoporosis and other bone diseases, including bone cancer; similarly, new diagnostic means have been proposed by this approach [26]. Conjugation of BPs to antibacterial agents has revealed promising significance in the cure of osteomyelitis, an acute or chronic bone infection [27,28,29]. 

Also the conjugation of BPs to different polymers has been evaluated as a bone targeting solution [30,31]. For instance, the amino-BP alendronate (ALE; Fosamax^®^) has been co-conjugated together with an anticancer agent to hydroxypropyl methacrylate (HPMA). With this system, a double strategy was looked for: a passive targeting *via* extravasation of the nanoconjugate from tumor vessels (EPR effect), and a concomitant active targeting to bone tissues due to the affinity of the polymer-conjugated ALE for HA [32].

### 1.2. A New Targeting Strategy

Targeted DDS can be advantageous compared to drug-BP prodrugs and conjugates for many reasons: a better drug protection from biodegradation in the bloodstream, a longer circulation time, a higher drug loading efficiency, scalable properties (particle size, surface charge, *etc*.), and other. Therefore, in a recent research project developed in our labs, we tried to merge the two “classical” strategies reported in Figure 1 as A and B, proposing an innovative approach for the targeting of bioactive compounds to the bone. Our assumption was to create a biocompatible nanocarrier showing affinity to the bone tissues (that is, an osteotropic nanocarrier), which in the meantime can easily encapsulate compounds active against bone diseases, such as antitumor, antibiotics, anti-osteolytic or anti-angiogenic drugs.

**Figure 2:**
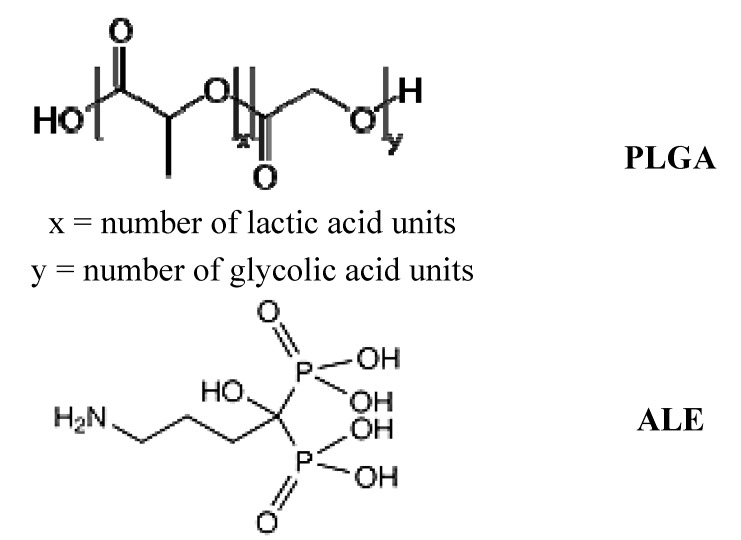
Chemical structure of Resomer^TM^ RG 502 H (PLGA) and alendronic acid (ALE).

In a first phase of the research, we produced a new polymeric biomaterial, possessing bone-seeking features, by conjugating a poly(lactide-*co*-glycolide) (PLGA) to the amino-BP ALE. PLGA copolymers are among the more common and accepted biocompatible and biodegradable materials proposed to realize controlled DDS [33]. Specifically, a 50:50 PLGA copolymer (Resomer^TM^ RG 502 H; Figure 2) was chosen, since the presence of a free carboxyl end group would easily allow the linkage of the BP.

ALE (4-amino-1-hydroxybutyldiene-1,1-phosphoric acid, Figure 2) is an amino-BP, clinically approved for the prevention and treatment of osteoporosis, glucocorticoid-induced osteoporosis, and therapy of Paget’s disease [34]. The choice of ALE was mainly linked to the presence of a free amine group that can be covalently linked to the PLGA carboxyl end-group without compromising the affinity for HA [35]. Moreover, ALE free acid form, occurring for the above reaction, can be easily prepared from the commercially available sodium salt. The preference for an amide bond between the targeting BP and the polymer backbone was also related to its relatively high resistance to enzymatic hydrolysis in the bloodstream, which should guarantee that the intact PLGA-ALE conjugate would arrive at its target.

In summary, as a first pace in the evaluation of the newly synthesized PLGA-ALE conjugate, its hemocompatibility and cytocompatibility were proven [36]. Afterwards, a nanoparticle carrier (NP) was produced starting from this new material, and the technological properties and biocompatibility of these NP were examined [37]. An anticancer agent, doxorubicin (DOX), was finally encapsulated in the PLGA-ALE NP and tested for its *in vitro* and *in vivo* activity [38].

## 2. Experimental Section

### 2.1. Synthesis and Characterization of the PLGA-ALE Conjugate

Poly(D,L-lactide-*co*-glycolide) (50:50) containing a free carboxylic acid end-group [Resomer^TM^ RG 502 H; inherent viscosity: 0.16–0.24 dL/g (0.1% in chloroform, 25 °C)] was purchased from Boehringer Ingelheim (Ingelheim am Rhein, Germany). Sodium alendronate (Sigma) treated with 5% aqueous acetic acid to form the free acid form; the white solid was filtered off, washed with water and freeze-dried. All reactants and solvents were purchased from Sigma-Aldrich Chimica srl (Milan Italy) and were used without further purification.

The PLGA-ALE conjugate was synthesized through two different procedures: in one attempt, a solution of the Resomer^TM^ in dry DMSO and dry dichloromethane (DCM) (1:1, v/v) was activated for 2 h at 0 °C using N'-(3-dimethylaminopropyl)-N-ethyl carbodiimide hydrochloride (EDAC), in the presence of 1-hydroxy-benzotriazole and triethylamine. Alendronic acid was dissolved in dry DMSO and added to the above reaction mixture, kept under magnetic stirring for 2 h at 2 °C and then at room temperature for about 8 h. The solvent was partially removed under high vacuum and the remaining solution was purified by dialysis against water (CelluSep H1 MWCO 2000; M-Medical srl.: Cornaredo, Italy). 

By an alternative method, the PLGA polymer was initially activated using N-hydroxysuccinimide (NHS) and dicyclohexylcarbodiimide in anhydrous dioxane at 15 °C under stirring for 3 h [39]. After removal of the produced dicyclohexylurea, the solution was poured in anhydrous diethyl ether, the solvent was decanted and the oily residue was purified by dissolution in anhydrous dioxane and re-precipitation with anhydrous diethyl ether (3 times), and finally freeze-dried. A solution of NHS-PLGA in dry DMSO was added of triethylamine and sodium alendronate and stirred for 12 h at room temperature. The solvent was then partially removed in vacuo and the concentrated solution was purified by dialysis against water, as above described. The dialyzed samples were frozen with liquid nitrogen and freeze-dried. 

Both methods gave analogous production yields (around 70–75%), a similar substitution degree of ALE (around 30% by mass analysis) and purity of the final conjugate; the first method was thus chosen for the further studies, as the more direct and simpler one. The chemical structure of the PLGA-ALE conjugate was confirmed by MALDI-TOF MS and ^1^H-NMR analyses; details can be found in the original article [36]. 

**Figure 3:**
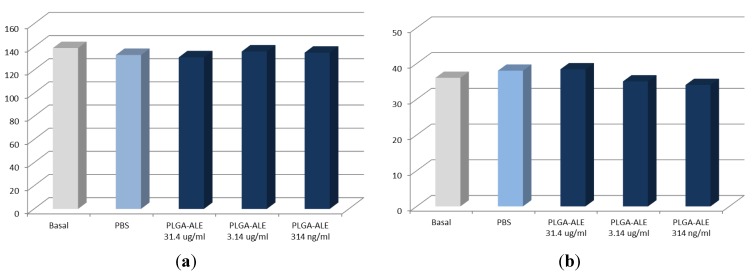
*In vitro* prothrombin activity (**a**) and activated partial thromboplastin time (APTT) values (**b**) of human plasma incubated with different concentrations of the PLGA-ALE conjugate (modified from reference [36]).

The blood and cyto-compatibility of the produced PLGA-ALE conjugate was assessed *in vitro*, to check any negative effect which might have hampered the production of NP and further biological studies. The induction of hemolysis of the conjugate had to be initially excluded, since red blood cells are among the first ones that come into contact with any injected product. Our experimental findings confirmed the absence of any hemolytic effect of the PLGA-ALE conjugate. Furthermore, human plasma samples incubated with PLGA-ALE at different dilutions showed a prothrombin activity and activated partial thromboplastin time (APTT) values not significantly different from control (incubation with PBS) (Figure 3a,b) [36]. These two biochemical parameters respectively evaluate the intrinsic and extrinsic phases of the formation of fibrin clots.

**Figure 4:**
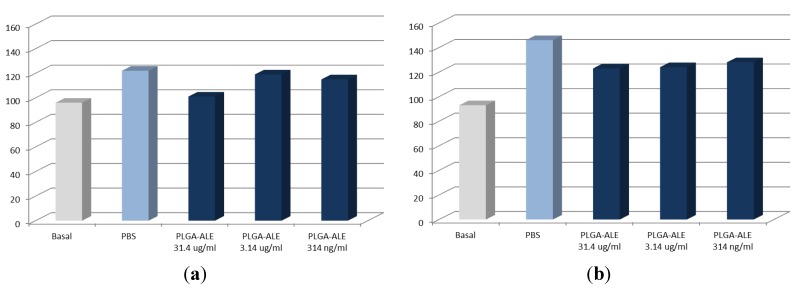
Viability of human umbilical vein endothelial cells (HUVEC) (**a**) and human primary osteoblasts from trabecular bone (**b**) after incubation with different concentrations of the PLGA-ALE conjugate (modified from reference [36]).

In the systemic circulation an injected material also comes across blood vessel endothelium before reaching the interstitium and surrounding tissues. The effect of PLGA-ALE on endothelial cells was therefore evaluated as a mean to verify the absence of cytotoxicity. The conjugate confirmed not be toxic for human umbilical vein endothelial cells (HUVEC), as proven by the neutral red test (Figure 4a). Lack of any toxicity was also demonstrated on human primary trabecular osteoblasts, in turn chosen as a model of target bone tissue for this polymer (Figure 4b) [36].

In conclusion, these preliminary studies proved that the new PLGA-ALE conjugate does not induce hemolysis on human erythrocytes, alterations of the plasmatic phase of coagulation, or any cytotoxic effect on endothelial cells and trabecular osteoblasts. 

### 2.2. PLGA-ALE Nanoparticles

An emulsion/solvent evaporation technique was followed to produce the NP [37]. Briefly, the conjugate was dissolved in either acetone, DMSO or a 1:1 (v/v) mixture of these solvents. The organic solution was added drop wise into a phosphate buffered saline solution (PBS), pH = 7.4, containing Pluronic F68. After stirring at room temperature for 10 min, the solvent was partially removed at 30 °C *in vacuo* and the concentrated suspension was purified by extensive dialysis against water. The choice of DMSO was made because of the relatively low solubility of the conjugate in acetone (with consequent low NP yields). However, neither DMSO was an ideal solvent, since the NP obtained using pure DMSO showed larger sizes compared to those obtained by dissolving the polymer in an acetone/DMSO mixture. 

All these NP showed an average size around 200–300 nm and a polydispersity index (PDI) of about 0.3, proof of a homogeneous distribution (Table 1). This size range appears to be extremely promising in the view of the further development of the proposed system as an injectable formulation. In a preliminary phase of this study, an alternative dialysis method for NP production was attempted [37], but it gave much larger particles (around 400 nm) (Table 1) and was not further exploited.

**Table 1:** Properties of PLGA-ALE NP prepared by the solvent evaporation method (using different solvents) or by a dialysis method.

Production method	Zeta Potential (mV)	Mean Particle Size (nm)	PDI
*Solvent evaporation (organic phase)*			
Acetone	−37.6 ± 4.1	188.0 ± 21.3	0.258
DMSO	−38.9 ± 3.7	286.9 ± 9.60	0.156
Acetone/DMSO (1:1, v/v)	−37.2 ± 5.0	198.7 ± 17.3	0.348
*Dialysis*	−39.3 ± 4.7	438.6 ± 11.1	0.033

**Figure 5:**
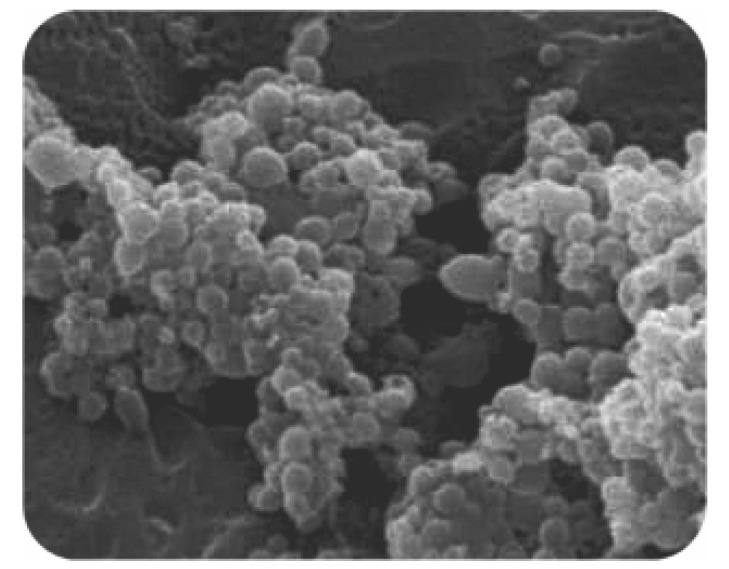
Scanning electron microscopy detail of a PLGA-ALE NP specimen prepared using 1:1 acetone/DMSO as the organic phase.

The NP showed a negative surface charge, with a mean ζ-potential around −40 mV, comparable to the value measured for the NP made of pure PLGA. Microscopy analysis showed spherical uniform particles having a smooth surface (Figure 5). It is noteworthy that these NP can be sterilized by gamma ray treatment with negligible particle size changes [37].

**Figure 6:**
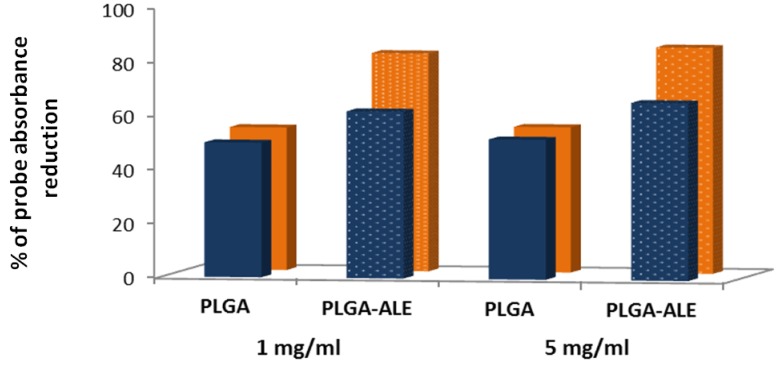
*In vitro* interaction studies between PLGA-ALE or PLGA NP and hydroxyapatite (HA). Oil Red O-loaded NP suspensions were incubated at r.t. for either 15 (blue bars) or 30 min (orange bars) with an aqueous suspension containing 5 mg/mL of HA. Affinity has been expressed as the percentage of probe absorbance decrease (at 523 nm) compared to the corresponding NP incubated without HA (modified from reference [37]).

The affinity of PLGA-ALE and PLGA NP for HA was also measured. For these experiments, the NP were loaded with a lipophilic colored probe (Red Oil O) and incubated with 1 or 5 mg/mL of HA, for either 15 or 30 min. As Figure 6 shows, PLGA-ALE NP had a higher affinity for HA compared to pure PLGA NP, which however displayed a basal physical interaction with the phosphate salt. Moreover, NP affinity appeared to increase proportionally with the incubation time and the concentration of HA in the suspension. 

The biocompatibility of PLGA-ALE NP was evaluated by different *in vitro* tests; they were chosen among those representative of the different biological systems that can come in contact with a material when injected systemically. The use of NP for drug delivery necessitates an accurate assessment of their biocompatibility [40,41]. For their nanoscale size, NP may have a reduced blood compatibility in comparison with the starting material: even if the biocompatibility of a macromolecule is well-established, the enormous increase of its surface when in the form of NP may bring on negative effects that are not given by the bulk material. 

### 2.3. Biocompatibility Studies on PLGA-ALE NP

The interactions between blood components and biomaterials are complex processes that can implicate erythrocyte and leukocyte damage, the activation of platelet and complement, and clotting. Polymeric materials and NP should not activate platelets and the plasmatic phase of coagulation, thus inducing thrombogenesis. As well, they should not lower the concentration of the plasmatic factors of coagulation, to avoid hemorrhagic events. Furthermore, in the view of targeting to the bones, the NP should not modify osteoblasts functionality. The PLGA-ALE NP were therefore assessed for their general hemo- and cytocompatibility [37]. 

**Figure 7:**
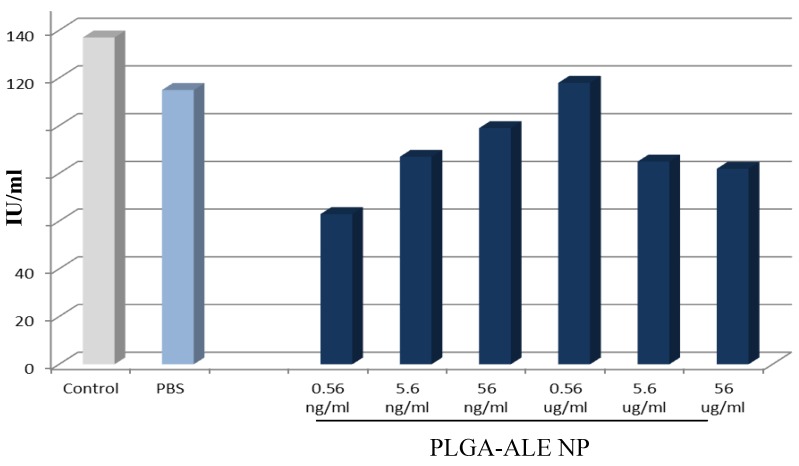
Concentration changes of platelet factor 4 after the incubation of PLGA-ALE NP with human blood (modified from reference [37]).

The first positive result was that they did not induced hemolytic effects on human erythrocytes (Table 2). Similarly, PLGA-ALE NP did not alter the total leukocyte number and their subpopulation distribution. Finally, they did not cause platelet adhesion or activation, as assessed by the measurement of platelet factor 4 release (Figure 7), a process that can trigger thrombotic phenomena after NP injection [37]. 

**Table 2:** *In vitro* hemolytic activity of PLGA-ALE NP (mean ± S.E. from 6 experiments). NP concentration was expressed as the amount of ALE in each sample (adapted from reference [37]).

Sample	NP concentration	% hemolysis
PLGA-ALE NP	0.56 ng/mL	0 ± 0.155
	5.6 ng/mL	0.167 ± 0.191
	56 ng/mL	0.356 ± 0.309
	0.56 μg/mL	0 ± 0.272
	5.6 μg/mL	0.289 ± 0.320
	56 μg/mL	0.001 ± 0.165
Saponin	-	132.7 ± 2.74
PBS	-	0
Distilled water	-	100

**Figure 8:**
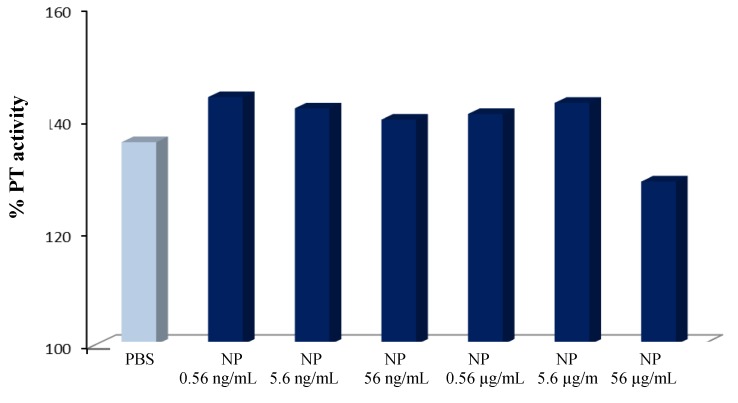
Variations in prothrombin activity given by different concentrations of PLGA-ALE NP.

The effects of PLGA-ALE NP on the plasmatic phase of coagulation and on complement components were also tested. It is known that a decrease of the coagulation factors levels may ease the hemorrhagic phenomena, while an increase of their activity can be responsible for thrombotic processes. In our experiments, only the highest tested concentration of PLGA-ALE NP led to a decrease of prothrombin activity, whereas lower concentrations increased it, but always within a range of normal values (Figure 8). Since also APTT (*i.e.*, the intrinsic and the common phase of coagulation) was not significantly affected by the NP (not shown), the observed changes could be due to an alteration of Factor VII of the extrinsic pathway of coagulation. Probably, Factor VII was adsorbed on the NP surface at high concentration and was less available for coagulation; conversely, lower concentrations of PLGA-ALE NP could activate factor VII, in a way similar to the effect of tissue factor. Another hypothesis could involve the activation of Factors XII and XI by the NP, similarly to silica gel or glass ([37] for more details).

Activation of complement induces the production and release of small biologically active peptides. C3a and C5a are chemo-attractive for leukocytes and promote their aggregation. Moreover, C5a induces the adhesion of granulocytes and monocytes to endothelia, their migration to the external tissues, enzyme release and production of pro-coagulant or platelet aggregating agents. C3a and the C5b-9 complex can directly activate platelets. Thereby, complement activation should not be considered as a strictly local phenomenon, but also produces systemic effects. It may follow either a classical and alternative pathways. In the classical pathway, the protein C1q recognizes and binds activators (usually immune complexes). The activation through the alternative pathway initiates by C3b binding to the surface of activator (e.g., microbial polysaccharides or lipids or surface antigens shown by viruses and cancer cells), thereafter tracking the same events of the classical pathway. When NP have been shown to activate the complement, this predominantly occurred by the latter pathway. 

**Figure 9:**
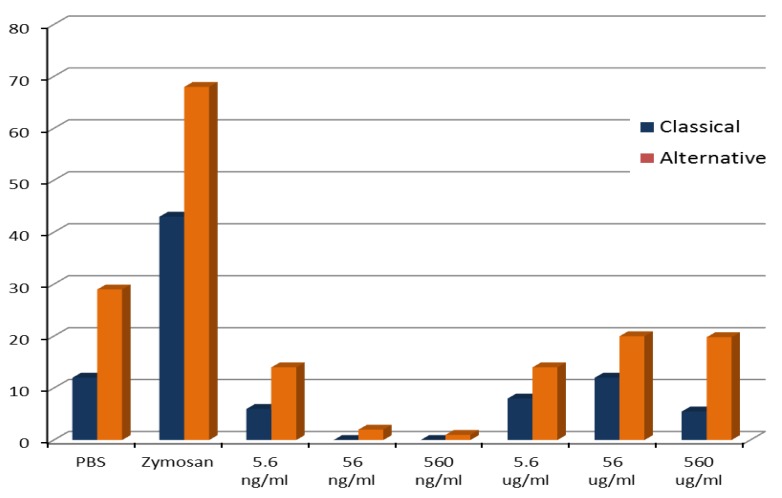
Percentage consumption of human serum complement activity via the classical or alternative pathways after incubation with different concentrations of PLGA-ALE NP.

**Figure 10:**
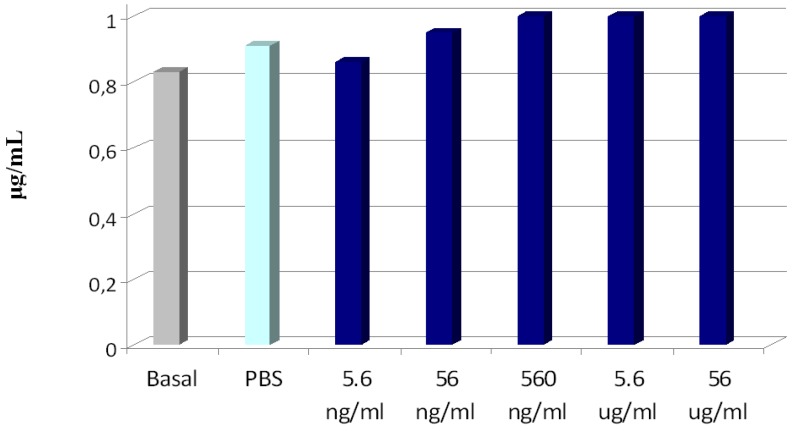
Bb fragment concentration after incubation with the PLGA-ALE NP.

The tested PLGA-ALE NP did not activate complement by either of the two pathways (Figure 9); the positive control zymosan induced a high complement consumption. Also the Bb fragment, which is produced during the complement activation by the alternative pathway, was not significantly affected by the PLGA-ALE NP (Figure 10).

**Figure 11:**
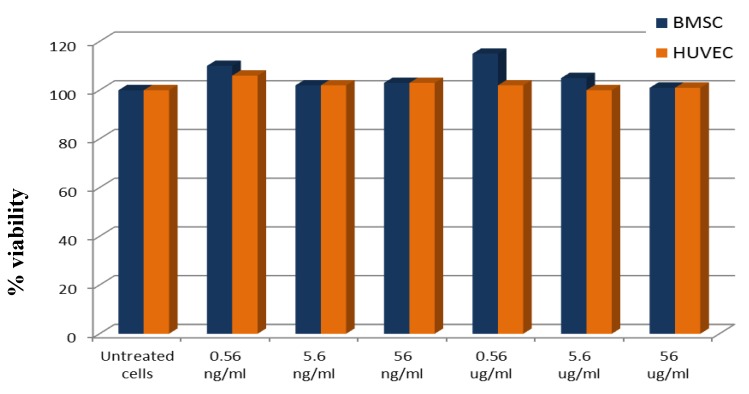
Viability assay of osteoblasts (BMSC) and endothelial cells (HUVEC) incubated with different concentrations of PLGA-ALE NP.

When injected in the bloodstream, NP rapidly came in contact with vessel endothelium before passing to the outer tissues; therefore, absence of damage to endothelial cells must be ensured. Moreover, bone oriented NP should not affect the vitality and function of normal osteoblasts. The cytotoxicity of the prepared PLGA-ALE NP was excluded on both endothelial (HUVEC) cells and osteoblasts derived by bone marrow stromal cells (BMSC). Cell viability was always higher than 80% upon 24 h-exposure to the various concentrations of NP or to PBS. Phenol, used as a positive control, reduced the cell viability to 19.0% (HUVEC) and to 27.5% (BMSC) (Figure 11).

In conclusions, our experiments demonstrated that the NP made of PLGA-ALE conjugate did not affect platelets, leukocytes and complement, did not induce hemolysis, and were not cytotoxic for osteoblasts and endothelial cells [37].

Furthermore, to confirm whether the PLGA-ALE NP retained the antiosteoclast properties of the parent BP, osteoclast cultures obtained from human peripheral blood mononuclear cells (PBMC) were incubated with PLGA-ALE NP at an equivalent ALE concentration of 0.64 μM or 6.4 μM, using free ALE as a positive control. Our experiments showed that, even conjugated with PLGA and in the form of NP, ALE retained the ability of inhibiting the osteoclast-mediated degradation of type I human bone collagen, inducing apoptosis in osteoclast cultures and causing a dose-dependent reduction of the number of osteoclasts. A detailed discussion of these experiments can be found in reference [42]. 

## 3. DOX-Loaded Nanoparticles

### 3.1. Preparation and Characterization of the NP [38]

The PLGA-ALE conjugate (9.8 mg) and DOX (doxorubicin hydrochloride, Sigma) (0.2 mg) were co-dissolved in 0.5 ml of acetone/DMSO (3:2, v:v). The organic phase was slowly dropped in 10 mL of PBS, pH = 7.4, containing 3 mg of Pluronic F68 (Fluka). After mixing at 13,500 rpm for 10 min, acetone was removed at 30 °C under reduced pressure, while DMSO was eliminated by dialysis against water (CelluSep H1 MWCO 2000; M-Medical Srl, Milan, Italy). The final volume of the suspension was adjusted with PBS. The obtained DOX-loaded NP showed a mean particle size (by PCS) of 245 nm (PDI = 0.250) and a net negative surface charge (Zeta potential = −26.50 mV), that was slightly lower that the value registered for the unloaded PLGA-ALE NP (around −38 mV; see Table 1). 

To calculate the DOX encapsulation efficiency, the NP suspension was dialyzed against water (see above) at room temperature for either 24 or 48 h. Samples were then ultracentrifuged at 15,000 rpm for 1 h and at 4 °C; the pellet was then frozen in liquid nitrogen and freeze-dried. A weighed amount of lyophilized sample was dissolved in DMSO, filtered (0.45 µm) and the absorbance measured by UV spectrophotometry at 480 nm. The encapsulation efficiency was calculated as the percent ratio of the amount of DOX incorporated in the NP to the initial amount used. An undialyzed NP sample was submitted to the same procedure to obtain the initial drug loading. Results showed that, regardless the dialysis time, DOX-loaded NP gave an analogous drug encapsulation (around 60%). This suggests that the aliquot of loaded drug was tightly associated with the polymer network.

To ascertain a mid-term physical stability, lyophilized NP specimens were stored in tight closed vials at room temperature and at −4 °C. After two months, no significant change in the mean particle size and Zeta potential values were measured. Finally, for the following biological studies, the NP were sterilized by gamma radiation (Gammatom, Como, Italy), and further characterized for size, PDI, Zeta potential and drug encapsulation efficiency, that did not show significant changes compared to the initial values.

### 3.2. *In Vitro* Studies

DOX is an anthracycline antibiotic, showing potent antineoplastic effects against leukemia and Hodgkin’s lymphoma, multiple myeloma, metastatic breast cancer, and many other cancers (prostate, thyroid, bladder, stomach, lung, ovary, soft tissue sarcoma). Its use is however hampered by the strong cardiac and bone marrow toxicity. DOX effectiveness has been greatly improved using passive targeting strategies, such as loading in liposomal carriers (*cf.* Doxil^®^/Caelyx^®^ or Myocet^®^) [43,44].

We encapsulated DOX in the PLGA-ALE NP and the antineoplastic effects of the carrier system were evaluated both *in vitro* on human cell lines, and *in vivo* on a mouse model of skeletal metastases from breast carcinoma [38]. A panel of potential DOX target cells was used in these studies: Saos-2 and U-2 OS osteosarcoma cell lines, SH-SY5Y neuroblastoma cell line, MDA-MB-231 and MCF7 breast adenocarcinoma cell lines, and ACHN renal adenocarcinoma cell line. All these tumor histotypes are representative of primary bone tumors or tumors that metastasize to bone. 

Treatment of cells with free DOX or DOX-loaded NP induced a similar, significant growth inhibition in most of the cell lines tested, especially at the highest doses. 

**Figure 12:**
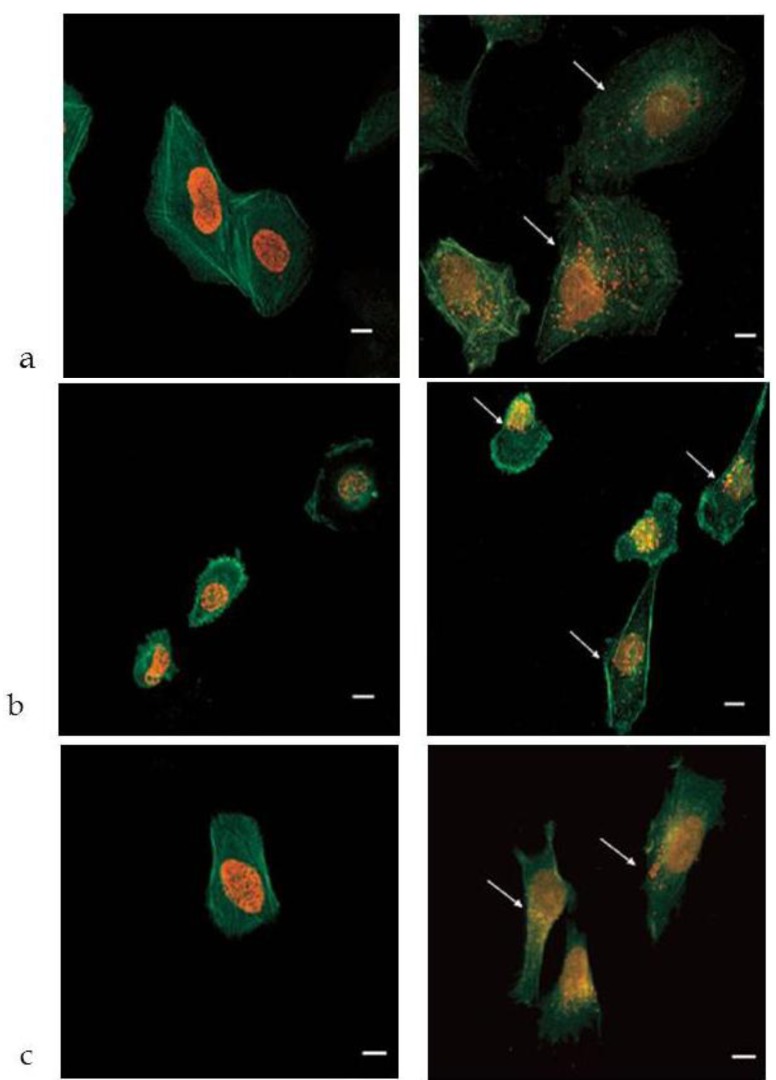
Confocal microscopy analysis of the cellular uptake of DOX. Free DOX (left) or DOX-loaded PLGA-ALE NP (right) were incubated for 24 h with U-2 OS cells (**a**), MDA-MB-231 cells (**b**), or SH-SY5Y cells (**c**). Cytoplasm fluorescence (red spots) is signaled by the arrows. Bars, 10 μm; magnification, 60X (arranged from reference [38]).

The intracellular accumulation and distribution of DOX-loaded NP was measured by confocal and fluorescence microscopy, as well as by electron microscopy. DOX intercalates into DNA and inhibits the progression of the topoisomerase II enzyme, which relaxes supercoils in DNA for transcription [45]. Due to the above mechanism of action, the localization of the drug in cell nucleus is of paramount relevance. Incubation of free DOX with the cells led to its complete accumulation in the nucleus, while the red fluorescence was totally absent in the cytosol (Figure 12, left pictures). Conversely, the cells treated with DOX-loaded NP showed some fluorescent spots also in the cytoplasm, most probably inside vacuoles (Figure 12, right pictures). Polymeric NP are in fact known to accumulate in lysosomal vesicles, from which the drug can be slowly released into the cytosol [46,47]. This phenomenon can explain the observed cytoplasmic fluorescence and insinuates that DOX-loaded NP were trafficked through the endo-lysosomal compartment. As an indirect confirmation of the above hypothesis, cells were incubated for longer times (48 or 72 h) with DOX-loaded PLGA-ALE NP, obtaining a cell growth inhibition profile close to that one given by free DOX [38]. 

TEM analysis (not shown) indicated for the MDA-MB-231 and Saos-2 cells treated with the DOX-loaded PLGA-PEG NP a dose-dependent intracellular accumulation of nano-sized structures (around 200-300 nm), often clustered and that could be easily discriminated from other typical intracellular vesicles.

### 3.3. *In Vivo* Studies

DOX-loaded NP were then tested in a mouse model of bone metastases [38]. The free drug and blank (unloaded) NP were administered in control experiments. Osteolytic lesions were induced by intratibial inoculation of the human breast carcinoma cells MDA-MB-231, which can induce bone metastases. Histological analysis confirmed the absence of evident abnormalities in different organs, suggesting the absence of general toxicity due to the drug-loaded carrier. 

**Figure 13:**
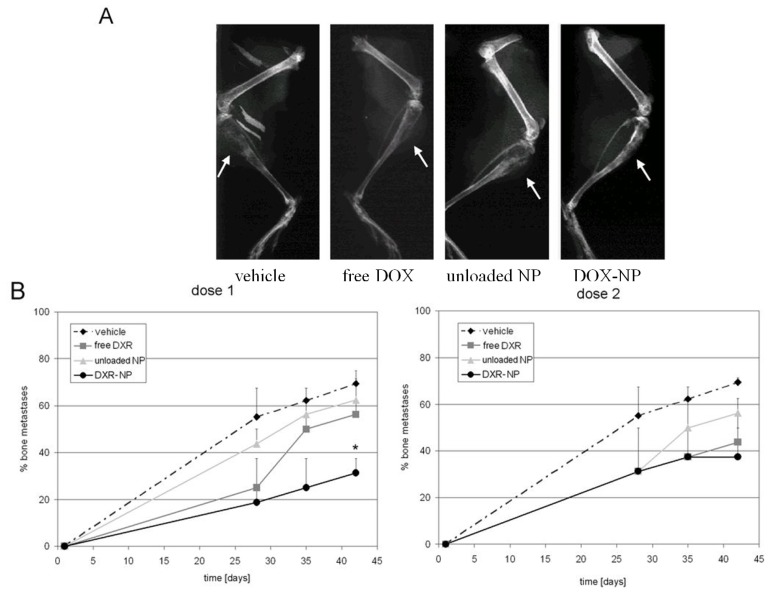
*In vivo* effect of DOX-loaded NP on the incidence of osteolytic bone metastases. BALB/c-nu/nu mice were injected intratibially with a suspension of MDA-MB-231 cells. Mice were treated weekly for six weeks with PBS, free DOX, blank NP, or drug-loaded NP (DOX-NP) with a dose of 0.2 or 1 mg/kg. (**A**) X-ray of hind limbs at the 42nd day after treatment of MDA-MB-231-injected mice and treated with PBS, free DOX, unloaded NP or DOX-NP at an equivalent drug dose of 0.2 mg/kg (osteolytic areas are evidenced by arrows). (**B**) incidence of osteolytic bone metastases after the same treatments for the lower (left graph) and the higher dose (right graph). Reported values are the mean ± SE of eight experiments. *p = 0.028 *vs.* vehicle (adapted from reference [38]).

Control mice treated with PBS developed pronounced osteolytic lesions detectable by X-ray analysis starting from the 28th day after the tumor cell inoculation (Figure 13A). All treatments were able to delay the onset of metastases and to reduce their incidence. In detail, blank NP displayed only a modest activity; it can be related to an inhibitory effect of ALE conjugated to PLGA on osteoclast activity that ultimately reduced tumor expansion, and maybe also to a direct effect of the BP on tumor cells [48]. This kind of synergism justifies why amino-BP have been recently used, alone or in combination with anticancer drugs, in the palliative treatment of patients with bone metastases from prostate and breast carcinomas [49]. 

Compared to free DOX (Figure 13B), loading the drug in the NP did not affect the drug efficacy on bone metastases formation and, moreover, at the lowest administered dose the carrier reduced the incidence of metastases to a significantly higher extent than the free drug (Figure 13B, left panel). The enhanced efficacy of DOX when loaded in the osteotropic carrier was an indirect proof of the successful and specific delivery of the drug to the bones. 

**Figure 14:**
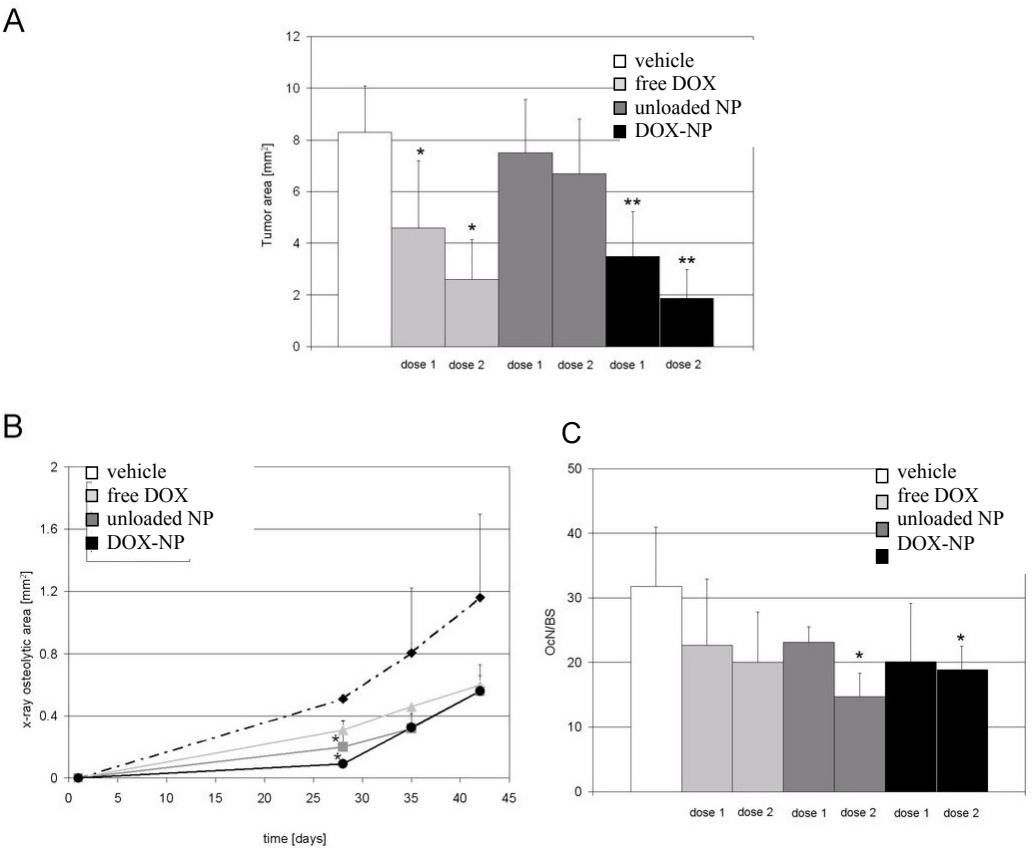
Effects of treatments on tumor area and osteolysis. Mice were treated with PBS, free DOX, blank NP, or DOX-loaded NP (DOX-NP) at an equivalent drug dose of 0.2 mg/kg (dose 1) or 1 mg/kg (dose 2). At the end point, the extension of the osteolytic area was determined by X-ray analysis; sections of the tibiae were histologically examined to evaluate tumor area and the number of osteoclasts on the bone surface. (**A**) tumor area (mean ± SD); (**B**) osteolytic areas quantified on X-ray images of mice treated with dose 1 (mean ± SE); (**C**) number of osteoclasts (OcN/BS) found on bone surface in TRAP stained sections of the tibiae (mean ± SD). *p < 0.05, **p < 0.005 (adapted from reference [38]).

The effect of treatments on the extension of tumor area, evaluated as the displacement of bone marrow by tumor cells was then assessed. Tumor size was significantly reduced in both animal groups that received either free DOX or DOX-loaded NP, at a drug concentration of 0.2 mg/kg or 1 mg/kg (Figure 14A). However, they induced a significant decrease of the osteolytic area only at the 28th day of the experiment. A similar trend of reduction of the osteolytic areas was registered in mice treated with either the free drug, blank NP and DOX-loaded NP. However, the latter produced a higher level of osteolytic inhibition than unloaded NP (0.090 ± 0.009 *vs*. 0.310 ± 0.040 mm^2^, p = 0.033). Both free and NP-loaded DOX attained a similar effect at the end of the test (day 42) (Figure 14B). 

Similarly, the histo-morphometric analysis of sections stained for TRAP activity showed a common trend in the reduction of the osteoclast number found at the bone surface at day 42 for all the treatments, although significant values were obtained only for treatments with unloaded or DOX-loaded NP at the highest dose, possibly because of a direct inhibitory effect of the conjugated ALE on osteoclasts (Figure 14C). 

## 4. Conclusions

All these experimental data provided *in vitro* and *in vivo* evidence of the effectiveness of a new osteotropic delivery system, made from a conjugate of PLGA and ALE. Loading DOX, and conceivably other antineoplastic drugs in such a carrier, may benefit from the synergism between the drug anticancer activity and ALE antiosteolytic activity to achieve a greater inhibition of tumor and metastases progression. Also drug loading in these biocompatible NP, allows a site-specific delivery of drugs to osteolytic areas, contributing to reduce their systemic side-effects. 

DOX loading in osteotropic PLGA-ALE NP did not affect the drug efficacy against bone metastases formation; the reduction of the incidence of metastases induced by DOX-loaded NP was significantly higher than that allowed by the free drug. Such results can be used as an indirect probe of the successful delivery of DOX to the bones and open the opportunity for further exploiting the potentiality of this novel osteotropic nanocarrier.
